# Children with Autism Spectrum Disorder in Times of COVID-19: Examining Emotional and Behavioral Problems, Parental Well-Being, and Resilience

**DOI:** 10.1007/s10803-022-05846-y

**Published:** 2023-05-22

**Authors:** Donna A. de Maat, Ruth Van der Hallen, Pieter F. A. de Nijs, Kirsten Visser, Dennis Bastiaansen, Femke L. Truijens, Elisabeth H. M. van Rijen, Wietske Ester, Peter Prinzie, Pauline W. Jansen, Linda P. Dekker

**Affiliations:** 1 Department of Psychology, Education, and Child Studies, Erasmus University Rotterdam, Burgemeester Oudlaan 50, Rotterdam, 3000 DR The Netherlands; 2 The Generation R Study Group, Erasmus MC University Medical Center Rotterdam, Rotterdam, The Netherlands; 3 Rotterdam Autism Consortium (R.A.C.), Rotterdam, The Netherlands; 4 Department of Child and Adolescent Psychiatry/Psychology, Erasmus MC University Medical Center Rotterdam, Rotterdam, The Netherlands; 5 Sarr Autism Rotterdam, Youz Child and Adolescent Psychiatry, Parnassia Group, Rotterdam, The Netherlands; 6 Yulius Center for Child and Adolescent Psychiatry, Dordrecht, The Netherlands; 7 Child and Adolescent Psychiatry, Curium-LUMC, Oegstgeest, The Netherlands; 8 Parnassia Bavo Group, Parnassia Bavo Academy, The Hague, The Netherlands

**Keywords:** COVID-19, autism spectrum disorder (ASD), emotional and behavioral problems, parental well-being, resilience

## Abstract

This longitudinal study assessed the impact of the COVID-19 pandemic on children with autism spectrum disorder (ASD; *n* = 62; *M*_age_ = 13 years) by measuring emotional and behavioral problems before and during the pandemic, and by comparing this change to a matched sample of children without ASD (*n* = 213; *M*_age_ = 16 years). Moreover, we examined whether indicators of parental well-being promoted resilience of children with ASD. Results showed that the mean change in problems did not differ between children with and without ASD. Importantly, some children showed an increase in problems, while others showed resilience. Parental well-being indicators were not related to resilience among children with ASD. The interindividual variability in responses, particularly among children with ASD, highlights the need for personalized support.

**Supplementary Information:**

The online version contains supplementary material available at 10.1007/s10803-022-05846-y.

The COVID-19 pandemic constitutes a challenging period for many families. In various countries, prevention measures were imposed, which led to drastic changes in life, particularly for families with children. During periods of lockdown, children could not physically attend school, parents had to work from home, and families were discouraged from having social contact with others. Previous findings indicate that the COVID-19 pandemic can result in higher levels of psychological distress among children, especially among those with neurodevelopmental disorders (Panchal et al., 2021) such as autism spectrum disorder (ASD). In fact, children with ASD may be particularly at risk due to their vulnerability to unpredictable and complex changes (Boulter et al., 2014; Demetriou et al., 2018).

Children with ASD have impairments in communication and reciprocal social interaction and exhibit restricted, repetitive patterns of behaviors or interests (American Psychiatric Association, 2013). As such, these children prefer highly predictable environments and may experience stress, anxiety, or confusion if unexpected changes occur (Baron-Cohen, 2006). The COVID-19 prevention measures may affect the functioning of children with ASD in particular due to the higher rates of co-occurring mental health problems (Lai, 2019), the disruption in day-to-day routines, and the reduced access to necessary supports. Simultaneously, some children with ASD may experience less stress during the pandemic as a result of reduced social interactions and demands (Ameis et al., 2020; Mumbardó-Adam et al., 2021), potentially improving their emotional and behavioral functioning (Mumbardó-Adam et al., 2021).

## Children with ASD During COVID-19

Some empirical studies indicated that children with ASD experienced more emotional and behavioral problems (EB-problems) during COVID-19, compared to before the pandemic (e.g., Colizzi et al., 2020; Di Renzo et al., 2020). However, other study findings revealed that children with ASD showed no changes between clinical scores (including anxiety and behavioral problems) collected at the beginning and the end of the lockdown period (Guidotti et al., 2020), although clinical scores before the pandemic were not assessed in this study. Mixed findings have also been found among other youth with pre-existing mental health problems, with some results suggesting an increase in problems over the course of the pandemic (Cost et al., 2022; Fischer et al., 2022), whereas other results point to stability or improvement in mental health during these challenging times (Bouter et al., 2021; Cost et al., 2022).

For now, it remains an open question whether children with ASD are more affected by the COVID-19 pandemic compared to children without ASD. Only a handful of studies have directly compared the impact of the pandemic on the functioning of children with and without ASD. These findings suggest that children with ASD experienced higher levels of EB-problems than children without ASD during the COVID-19 pandemic (e.g., Nonweiler et al., 2020; Polónyiová et al., 2022; Waite et al., 2021). In another study, youth with ASD showed more negative changes in behavior during the lockdown period than their peers without ASD, as retrospectively reported by parents (Amorim et al., 2020). Nevertheless, many children with ASD experience elevated problems in normal times as well (Lai et al., 2019), underscoring the need to consider children’s pre-pandemic functioning when comparing the impact of the COVID-19 pandemic between children with and without ASD. However, due to a lack of longitudinal studies measuring child behavior before the pandemic, it is still unclear how the COVID-19 outbreak has affected the functioning of youth with ASD as compared to youth without ASD.

## Resilience of Children with ASD

Despite recent studies on the functioning of children with ASD during the COVID-19 pandemic, little is known about why some children with ASD show more resilience than others during this period. Resilience refers to the dynamic process of relatively positive adaptation despite exposure to significant risk (Masten, 2018). Parental well-being may be an important factor that contributes to the resilience of children with ASD under stressful conditions, such as the COVID-19 pandemic. Previous findings indicate that better parental mental health, including lower levels of parental depression, anxiety, and stress, can protect children with ASD from emotional and behavioral difficulties during COVID-19 (Polónyiová et al., 2022) and promote children’s post-disaster mental health (Cobham et al., 2016). In addition, the extent to which parents feel socially connected to other people may play a role in children’s positive outcomes and resilience. Previous work suggests that perceived social support of parents is a key protective factor for children with ASD in normal times (Drogomyretska et al., 2020) as well as during the COVID-19 pandemic (Tokatly Latzer et al., 2021). Parents’ perceptions of social connectedness to others could diminish parental stress (e.g., Zaidman-Zait et al., 2017), which in turn may lead to more effective parenting practices (e.g., Keen et al., 2010) and fewer EB-problems among children with ASD during the lockdown (Chen et al., 2021; Polónyiová et al., 2022). Yet, the aforementioned studies examining the role of parental well-being in the resilience of children with ASD did not include a pre-pandemic assessment. To better understand which children are at risk and which show resilience to the COVID-19 situation, longitudinal studies are needed that focus on potential protective factors and simultaneously consider children’s functioning before the pandemic.

## The Current Study

The COVID-19 pandemic offers a unique opportunity to examine how challenging or stressful situations affect families from populations potentially at risk. Obtaining more knowledge on the impact of the pandemic on children with ASD and protective factors at the family level is crucial for providing tailored interventions and support for children with ASD and their parents. This knowledge may also be used to better assist vulnerable families during other life-changing events, such as following natural disasters or health crises. Therefore, the current study aimed to (1) investigate the impact of the COVID-19 pandemic on EB-problems of children with ASD as compared to children without ASD. Uniquely, we had pre-COVID-19 data on children’s functioning, which enabled us to determine the impact of the COVID-19 pandemic on children’s problems more objectively. Based on the available literature, we hypothesized that, on average, children with ASD experienced a larger increase in EB-problems from pre-COVID-19 (T0) to during COVID-19 (T1) than children without ASD.

Moreover, we aimed to (2) examine to what extent parental well-being, i.e., indicators of parental mental health and social connectedness, was associated with resilience of children with ASD in times of COVID-19. According to resilience frameworks (e.g., Infurna & Luthar, 2018; Masten, 2018; Rutter, 2012), resilience is never directly measured but instead is indirectly inferred based on the presence of two dimensions: exposure to *risk* that increases the probability of negative outcomes and relatively *positive adaptation* that is better than expected, given the risk experienced. In the current study, we operationalized *risk* as children’s exposure to the COVID-19 pandemic, whereas *positive adaptation* was assessed by better emotional and behavioral functioning, specifically a smaller increase in emotional and behavioral problems from pre-pandemic to during the pandemic. We expected that higher levels of parental mental health and social connectedness were related to smaller increases in problems from T0 to T1 among children with ASD, i.e., potentially contributing to resilience during COVID-19.

## Methods

Our study hypotheses, design, and analysis plan were preregistered; see https://osf.io/a3y67. Any deviations from the planned methodology are indicated throughout the manuscript.

### Participants and Procedures

This investigation was based on two studies. Families participating in the ASD & COVID-19 study (Dekker et al., 2022) were included for aims 1 and 2. Additionally, we used data of families participating in the Generation R Study (Kooijman et al., 2016) to make a comparison with children without ASD for aim 1. Both studies were approved by the Medical Ethics Committee of the Erasmus Medical Center. Informed consent was obtained from all participants.

### ASD & COVID-19 Study

**Fig. 1:**
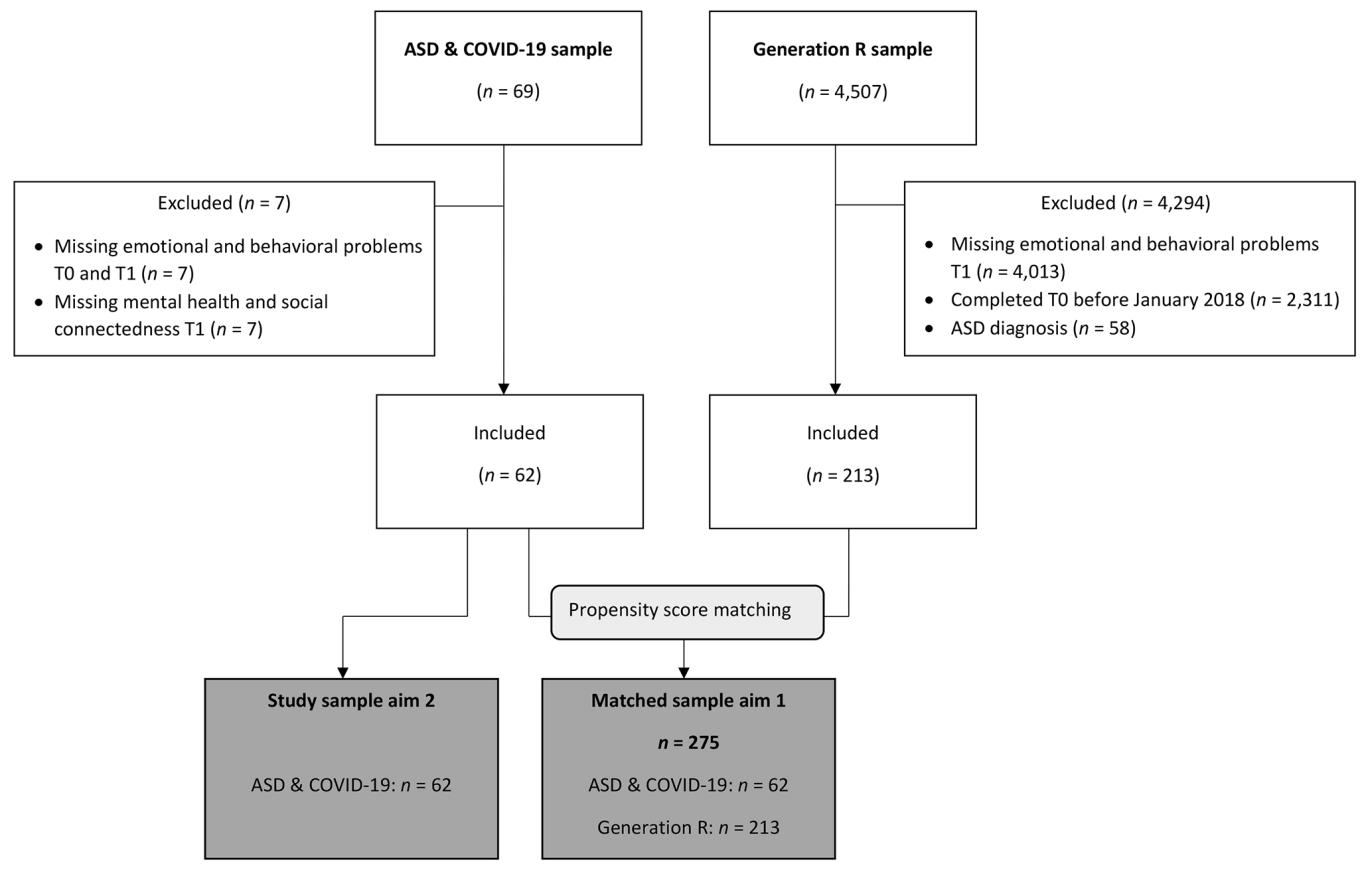
Participants’ inclusion and exclusion flow diagram

The ASD & COVID-19 sample contains 69 families in the region of Rotterdam, the Netherlands, with a child or adolescent diagnosed with ASD, who were in care in one of three specialized mental health care institutions (i.e., Youz, Yulius, or Erasmus Medical Center – Sophia Children’s Hospital). Children were diagnosed with ASD by a qualified mental health care professional. For more details on the recruitment and study design, see Dekker et al. (2022). One of the parents (89% mothers; 11% fathers) filled out an online survey on child and family functioning during the second COVID-19 lockdown in the Netherlands (for information on restrictions during the lockdown, see Supplementary Material). Data were collected between 11 January 2021 and 26 May 2021 (T1). Prior to any COVID-19 related restrictions, between 1 March 2019 and 1 March 2020 (T0), data about child functioning were collected as part of routine outcome monitoring by the participating institutions. In the current study, we included all families with data on child EB-problems at T0 or T1 *and* data on parental mental health or social connectedness at T1. This resulted in a sample of 62 children (see Table 1 for sample descriptives; see Fig. 1 for a flowchart of participants).

**Table 1:** Characteristics of Study Samples

	ASD & COVID-19 Matched *n* = 62	Generation R Matched *n* = 213	Generation R Full cohort *n* = 4,507
*Child characteristics*			
Age at T1, years	12.8 (4.5)	15.8 (0.6)	16.8 (1.0)
Sex			
Boy (%)	74.2	75.8	48.7
Girl (%)	25.8	24.2	51.2
Ethnicity			
Dutch (%)	75.6	58.6	66.4
Other (%)	24.4	41.4	33.6
IQ	90.5 (27.1)	104.5 (13.62)	103.1 (13.5)
SRS-2^a^	94.4 (32.1) range 21–158	-	-
*Parent characteristics* ^b^			
Age at T1, years	45.6 (7.4)	46.4 (6.4)	48.4 (5.6)
Sex			
Male (%)	11.1	-	-
Female (%)	88.9	-	-
Education level			
Primary (%)	3.1	2.7	2.4
Secondary (%)	50.0	34.1	33.9
Higher (%)	46.9	63.2	63.8
Marital status			
Married/living together (%)	72.2	81.0	89.2
Divorced/no partner (%)	27.8	19.0	10.8
Number of children	2.6 (1.7) range 1–12	2.0 (1.8) range 1–5	2.2 (0.8) range 1–6

^a^ The Social Responsiveness Scale (SRS-2; Roeyers et al., 2015) was completed by parents at T1 to assess the severity of children’s ASD symptoms

^b^ In Generation R, parental characteristics represent mothers’ age, education level, and marital status. As adolescents filled out the questionnaires at T0 and T1 in Generation R, the distribution of parental sex at those waves was unknown

### Generation R Study

Participants from the ASD sample were matched to controls from the Generation R Study, a prospective population-based cohort in Rotterdam, the Netherlands. The design and sample characteristics of the study have been described in detail elsewhere (Kooijman et al., 2016; Jaddoe et al., 2007). During the second lockdown in the Netherlands, all adolescents participating in the study were invited to participate in an online survey. Data were collected between 21 December 2020 and 3 March 2021 (T1). Prior to the pandemic, information about child functioning was available at the average age of 13 years (20 April 2016–30 January 2020; T0). In the current study, we included all adolescents with data available on child EB-problems at T0 and T1, who filled out the baseline questionnaire (T0) between 1 January 2018 and 30 January 2020 to better match the baseline period of the participants in the ASD & COVID-19 study (not preregistered), and without an ASD diagnosis. Of the 494 adolescents with data available on child EB-problems at T0 and T1, 213 adolescents completed T0 between 1 and 2018 and 30 January 2020. Within this group, no adolescents were diagnosed with ASD, resulting in a sample of 213 children.

To compare the ASD and Generation R samples, we created a matched dataset using propensity score matching in R (R Core Team, 2017) using the package *MatchThem* (Phisgar et al., 2020). We matched the full sample of 275 children (62 from ASD sample, and 213 from Generation R) on child sex and parental age. Following power analysis (Faul et al., 2009), sample sizes were found to be sufficiently large for the analysis of both aim 1 (*n* = 275, *f*^2^ = 0.15, α = 0.05, power > 0.99) and 2 (*n* = 62, *f*^2^ = 0.15, α = 0.05, power = 0.91). We performed attrition analyses and used multiple imputation to handle missing data. A detailed description of the study timeline (Figure S1), matching procedure, and missing data handling is provided in Supplementary Material.

### Materials

#### Child EB-Problems (T0 and T1)

In both samples, children’s EB-problems were measured by the Brief Problem Monitor (BPM; Achenbach et al., 2017) at T0 and T1. We chose to use the BPM, an abbreviated version of the Child Behavior Checklist (CBCL), to minimize burden on the participants. Previous work has supported the psychometric properties of the BPM and indicated an excellent correspondence between the BPM and CBCL total scores (e.g., *r* = .95; Piper et al., 2014; *r* = .93; Richter, 2015). Parent report (BPM-P) was used in the ASD sample and adolescent report (BPM-Y) was used in Generation R. All 19 items were answered on a 3-point scale (0 = *not true*, 1 = *somewhat or sometimes true*, 2 = *very true or often true*). We computed total problem scores for T0 and T1 by summing all item scores (range α = 0.77–0.87; theoretical range: 0–38), with higher scores indicating more EB-problems. In addition, we calculated difference scores, i.e., Δ EB-problems = EB-problems T1 – EB-problems T0. Positive scores on Δ EB-problems indicated an increase in child EB-problems from T0 to T1, whereas negative scores pointed to a decrease.

#### Parental Mental Health (T1)

In the ASD sample, parents rated the extent to which they had experienced anxiety and depression symptoms during the past week at T1 using the Anxiety (6 items) and Depression (6 items) subscales of the Brief Symptom Inventory (BSI; Derogatis 1993). Previous findings indicated good reliability and validity of the Anxiety and Depression subscales (e.g., Derogatis 1993; Recklitis et al., 2006) and demonstrated that these subscales measure one single dimension representing ‘psychological distress’ (Endermann, 2005; Khalil et al., 2011). Items were answered on a 5-point scale (ranging from 0 = *not at all* to 4 = *very much*). We reverse coded the scores so that higher scores indicated better mental health of parents. We computed total parental mental health scores by summing all items on the two subscales (α = .89; theoretical range: 0–48).

#### Social Connectedness (T1)

In the ASD sample, parents reported on their feelings of loneliness at T1 using 3 items (“How often do you feel alone?”; “How often do you feel left out?”; “How often do you feel isolated from others?”) of the Three-Item Loneliness Scale (Hughes et al., 2004), which has demonstrated good reliability and validity in previous work (e.g., Hughes et al., 2004; Klein et al., 2021; Trucharte et al., 2021). The loneliness items were answered on a 3-point scale (0 = *never or rarely*, 1 = *sometimes*, 2 = *often*). We included an additional item on social isolation that was completed by parents (“How socially isolated did you feel in the past 7 days”?), answered on a 10-point scale (ranging from 1 = *not socially isolated* to 10 = *extremely socially isolated*). Scores were reverse coded so that higher scores indicated fewer feelings of loneliness and social isolation, i.e., more perceived social connectedness. To combine the 4 items, we standardized (*z* scores) the items and calculated sum scores (α = 0.78; theoretical range: -16–16).

#### Covariates

We considered the inclusion of child age at T1 (years) and child IQ at T0 as covariates in the analyses. In the ASD sample, information on IQ was collected by trained practitioners using various intelligence scales, e.g., the Wechsler scales (Wechsler Intelligence Scale for Children – 5th ed.; WISC-V; Wechsler 2014; Wechsler Adult Intelligence Scale – 4th ed.; WAIS-IV; Wechsler 2008), or other comparable scales. In Generation R, IQ was assessed by trained researchers using the WISC-V (Wechsler, 2014). In case only mental age equivalents were available, these were transformed into estimations of full-scale IQ. Additionally, we considered including other background variables as covariates, based on our inclusion criteria (see Statistical Analyses).

### Statistical Analyses

#### Comparison Between Children with and Without ASD

First, to compare the impact of the COVID-19 pandemic in children with ASD versus without ASD (aim 1), we conducted a linear regression analysis in the matched sample (*n* = 275) with the type of sample (0 = control; 1 = ASD) as predictor and Δ EB-problems from pre-pandemic to lockdown as outcome variable. In addition, we computed the percentages of problem scores in the elevated range (≥ 93rd percentile; Achenbach et al., 2017) at T0 and T1 per sample. Next, we exploratively tested whether the variances for problem scores at T0 and T1 differed between the ASD and control samples (not preregistered).

#### Parental Well-Being and Resilience of Children with ASD

Second, we examined to what extent indicators of parental mental health and social connectedness were related to resilience of children with ASD in times of COVID-19 (aim 2) by performing a multiple regression analysis. This analysis was only performed in the ASD sample (*n* = 62), due to a lack of matching measures in the control sample. Parental mental health and social connectedness indicators were simultaneously included as predictors and Δ EB-problems as outcome variable. To interpret the results in terms of resilience, the direction of any significant regression coefficient (positive or negative) was evaluated. For example, a negative regression coefficient of parental mental health would indicate better parental mental health is related to smaller increases in child EB-problems from T0 to T1, i.e., potentially contributing to resilience. Finally, we conducted an exploratory logistic regression analysis (not preregistered) to test whether parental mental health and social connectedness were related to the direction of Δ EB-problems among children with ASD (0 = decrease; 1 = increase).

#### Effect Sizes and Covariates

We computed pooled standardized regression coefficients (β) and *R*^*2*^-values as measures of effect size (Van Ginkel, 2019). Covariates were included in the regression analyses if these variables correlated significantly with the relevant predictors (i.e., Model 1: sample; Model 2; parental mental health and social connectedness) and the outcome variable (i.e., Δ EB-problems). Based on these criteria, we included child IQ and EB-problems at T0 as covariates in Model 1, and we did not include any covariates in Model 2.

## Results

### Comparison Between Children with and Without ASD

**Fig. 2:**
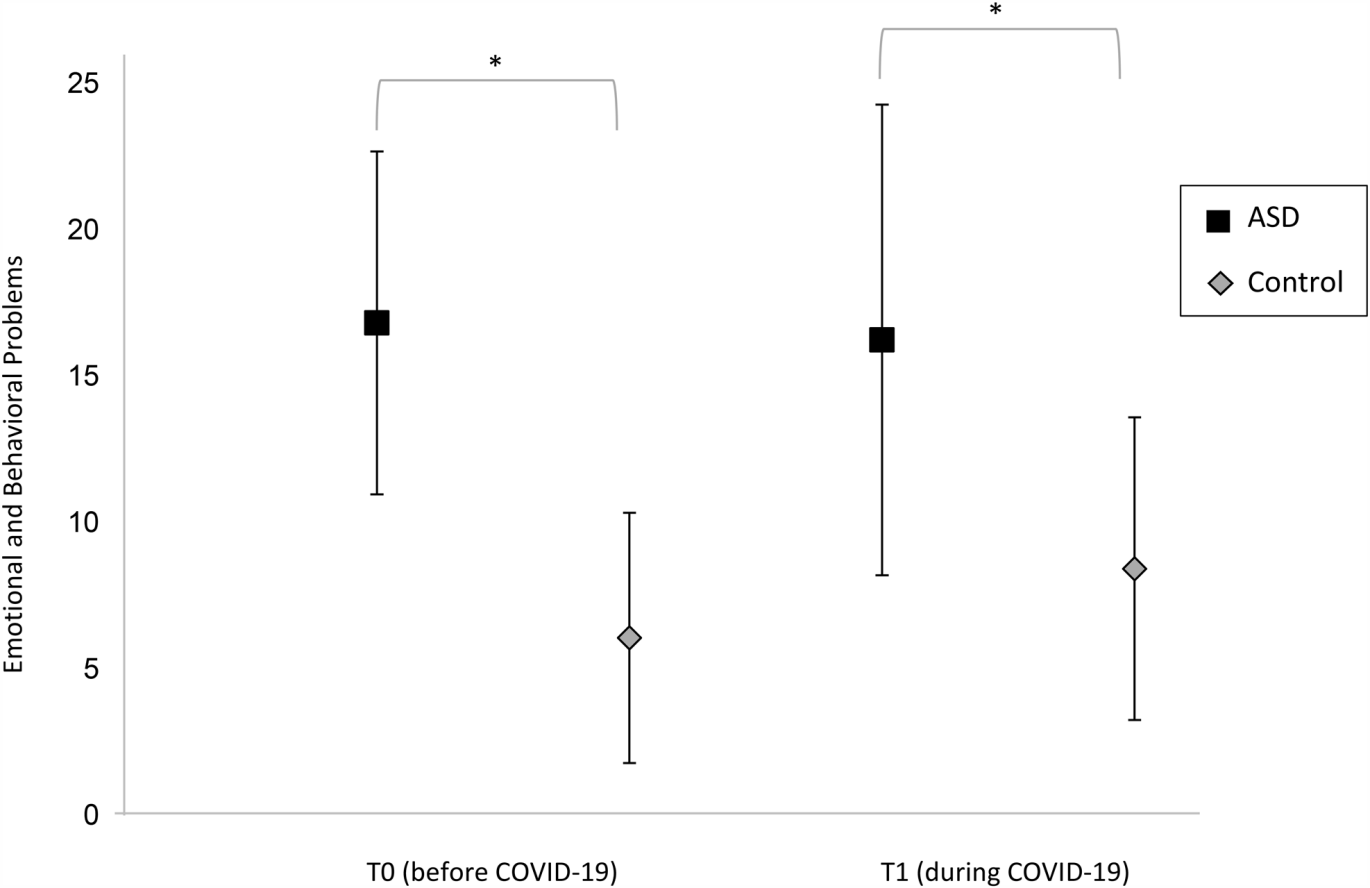
Means and standard deviations of child emotional and behavioral problems across samples. (Note: ASD = Autism spectrum disorder, * Levene’s test showed that the variances for child emotional and behavioral problems were significantly larger in the ASD sample than in the control sample at T0 (*F* (1, 273) = 3.90, *p* = .049) and T1 (*F* (1, 273) = 19.72, *p* < .001))

Descriptive statistics of the imputed study variables are shown in Table 2. Children with ASD scored higher on EB-problems than children without ASD both before (*t* (81) = -13.46, *p* < .001) and during the COVID-19 pandemic (*t* (76) = -7.24, *p* < .001), but the level of problems remained fairly stable. In the control sample, the children had higher EB-problem scores during the COVID-19 pandemic than before, as also visualized in Fig. 2. Regression analyses showed that Δ EB-problems did not differ significantly between children with versus without ASD, after controlling for child IQ and problems at T0 (*B*_ASD sample_ = 2.05, *SE* = 1.65, *p* = .222, β = 0.16). Higher levels of pre-pandemic problems at T0 were significantly associated with smaller increases in problems from pre-pandemic (T0) to lockdown (T1; *B*_problems T0_ = -0.34, *SE* = 0.09, *p* = < 0.001, β = − 0.42). Full regression parameters are displayed in Table 3.

**Table 2:** Descriptive Statistics and Spearman Correlations for Imputed Study Variables

Variable	1	2	3	4	5	6	7	8	*M*	*SD*	Min	Max
*ASD & COVID-19 (n = 62)*												
1. Δ EB-problems_c_^a^	-								0.3	4.6	-16.0	11.0
2. EB-problems_c_ T0	− 0.34*	-							16.8	5.9	1.7	33.0
3. EB-problems_c_ T1	0.22	0.32	-						16.3	8.0	1.0	36.3
4. Sex_c_^b^	− 0.24	0.26	0.12	-					1.3	0.4	-	-
5. Age_c_	− 0.17	0.17	− 0.15	− 0.02	-				12.8	4.5	5.9	22.1
6. IQ_c_	− 0.13	0.03	− 0.18	0.15	0.02	-			90.5	27.1	28.0	134.0
7. Social connectedness_p_^c^	0.01	0.02	− 0.04	− 0.08	0.08	− 0.08	-		0.0	3.1	-10.0	4.0
8. Mental health_p_	− 0.02	− 0.18	− 0.28*	− 0.22	0.33*	− 0.03	0.51***	-	42.6	5.3	24.0	48.0
*Generation R (n = 213)*												
1. Δ EB-problems_c_	-								2.4	4.9	-12.0	16.0
2. EB-problems_c_ T0	− 0.48***	-							6.1	4.3	0.0	24.0
3. EB-problems_c_ T1	0.54***	0.41***	-						8.4	5.2	0.0	31.0
4. Sex_c_	0.27***	0.05	0.33***	-					1.2	0.4	-	-
5. Age_c_	0.07	0.02	0.09	− 0.10	-				15.8	0.6	15.0	17.8
6. IQ_c_	0.45***	− 0.23**	0.24**	0.03	0.10	-			104.5	13.6	74.0	139.0

*Note*. EB-problems = emotional and behavioral problems. _c_ = child, _p_ = parent

^a^ Δ EB-problems = EB-problems T1 – EB-problems T0. Descriptives for imputed values are shown, therefore, the mean of Δ EB-problems does not equal the mean of EB-problems T1 minus the mean of EB-problems T0. ^b^ 1 = boy; 2 = girl. ^c^ Social connectedness based on standardized values

*p < .05. **p < .01. ***p < .001

**Table 3:** Regression Parameters of the Models Predicting Δ EB-Problems

	Δ EB-Problems				
	*B*	*SE*	*p*	β	*R* ^2^
*Model 1: matched samples (*n *= 275)*					0.21
ASD sample^a^	2.05	1.65	0.222	0.16	
IQ_c_	0.04	0.02	0.179	0.13	
EB-Problems T0_c_	**-0.34**	**0.09**	**< 0.001**	**− 0.42**	
*Model 2: ASD sample (*n *= 62)*					0.01
Mental health_p_	-0.02	0.15	0.901	− 0.02	
Social connectedness_p_	-0.04	0.25	0.878	− 0.03	

*Note*. EB-problems = emotional and behavioral problems. _c_ = child, _p_ = parent. Significant coefficients (p < .05) are displayed in bold

^a^ In model 1, type of sample was included as a dichotomous variable (0 = control; 1 = ASD).

In the ASD sample, 33 (53.2%) versus 30 (48.1%) children had elevated problem scores (≥ 93rd percentile) at T0 and T1, respectively, compared to 10 (4.6%) versus 27 (12.7%) children in the control sample. Besides, Levene’s test demonstrated that the variances for EB-problems were significantly larger in the ASD sample than in the control sample at T0 (*F* (1, 273) = 3.90, *p* = .049) and T1 (*F* (1, 273) = 19.72, *p* < .001), see Fig. 2. This result indicated that children with ASD showed more interindividual variability in EB-problems than children without ASD both before and during the COVID-19 pandemic.

### Parental Well-Being and Resilience of Children with ASD

Furthermore, the results indicated that indicators of parental mental health (*B* = -0.02, *SE* = 0.15, *p* = .901, β = − 0.02) and social connectedness (*B* = -0.04, *SE* = 0.25, *p* = .878, β = − 0.03) were not significantly related to Δ EB-problems in the ASD sample, i.e., did not contribute to resilience of children with ASD (see Table 3).

Finally, the exploratory logistic regression analysis yielded the same conclusions as our main analyses. The results revealed that indicators of parental mental health (*B* = -0.01, *SE* = 0.08, *p* = .881, β = − 0.06) and social connectedness (*B* = 0.01, *SE* = 0.13, *p* = .925, β = 0.04) were not significantly related to the direction (i.e., positive or negative) of Δ EB-problems in the ASD sample. Thus, levels of parental mental health and social connectedness did not differ between children who experienced an increase (*n* = 32) in problems and those who remained stable or experienced a decrease (*n* = 30).

## Discussion

The current study compared the impact of the COVID-19 pandemic on EB-problems of children with ASD and a matched control group, and examined potential predictors of resilience of the children with ASD. The results indicate that the change in EB-problems following COVID-19 did not differ significantly between the groups, after adjustment for child IQ and problem level before the pandemic. This result is important, as various studies reported that children with ASD experienced more EB-problems than peers without ASD during the pandemic (e.g., Nonweiler et al., 2020; Polónyiová et al., 2022; Waite et al., 2021). However, this was the first longitudinal study that directly compared the *change* in EB-problems from pre-COVID-19 to during COVID-19 between children with and without ASD. On average, children with ASD showed a high, yet stable level of EB-problems across T0 and T1, whereas children without ASD reported fewer problems at both timepoints, yet they slightly increased from T0 to T1. As recently suggested by others (Ameis et al., 2020; Mumbardó-Adam et al., 2021), it is likely that children with ASD benefitted from the COVID-19 pandemic related measures, as they may have felt less social pressure, exclusion, and rejection, which could result in reduced stress and improved functioning. In contrast, children without ASD may have been more negatively affected by the loss of face-to-face social contacts and activities, given the salience of peer relationships for youth’s psychological well-being (Orben et al., 2020) and evidence suggesting that children and adolescents without ASD are more engaged with peers and have higher friendship quality compared to ASD youth (e.g., Locke et al., 2010; Petrina et al., 2014).

Moreover, in line with previous work (e.g., Colizzi et al., 2020; Cost et al., 2022; Tokatly Latzer et al., 2021), our results point to interindividual variability in children’s responses to the COVID-19 situation within both samples: Some children showed an increase in EB-problems following the lockdown, whereas other children remained stable, or showed a decrease in problems. Children with ASD showed more interindividual variability in problems than children without ASD both before and during the COVID-19 pandemic, which emphasizes the notable heterogeneity within the ASD population (e.g., Georgiades et al., 2013) and highlights the importance of considering individual differences when examining the impact of the pandemic or other stressful situations in this clinical group (Cost et al., 2022).

Additionally, we examined to what extent parental mental health and social connectedness acted as protective factors for children with ASD in times of COVID-19. Our findings showed that indicators of parental mental health and social connectedness were not related to the change in child problems following the COVID-19 pandemic. This conclusion was supported by additional analyses which indicated that the mental health and social connectedness of parents did not differ between children who increased versus decreased in terms of problems. Hence, these parental characteristics were possibly not related to resilience in times of COVID-19. This result contrasts with previous findings suggesting that parents’ mental health (Polónyiová et al., 2022) and social support (Tokatly Latzer et al., 2021) may promote positive emotional and behavioral functioning of children with ASD during the COVID-19 pandemic. Several explanations are possible for the limited evidence supporting these parental characteristics as potential protective factors in this study. First, parents of children with ASD in the current study reported relatively high levels of mental health (i.e., low levels of anxiety and depression) and social connectedness (i.e., low levels of loneliness), which may have reduced the ability to detect any association of parents’ mental health and social connectedness with the change in children’s EB-problems. These characteristics may represent a sampling bias, as it is possible that parents that struggled more were less likely to participate. Second, children with ASD may be less affected by others, parents in this case, showing distress or fear than children without ASD (Dawson et al., 2004; Sigman et al., 1992). Third, we assessed these indicators of mental health and social connectedness by asking parents how they felt *in the past 7 days*. However, it is known that feelings of anxiety and depression can fluctuate over time (e.g., Schoevers et al., 2021). Perhaps more stable characteristics of parents, such as personality and coping style (Burlaka et al., 2019) or parenting practices (Oliveira et al., 2022), might have a greater impact on children’s functioning in the context of life-changing situations. Yet, more research on potential protective factors for child resilience to the COVID-19 pandemic including longitudinal assessments of parental characteristics is needed to test this hypothesis. Fourth, other factors that were not considered in the current study could have affected the functioning of parents and children during the pandemic. For instance, the severity of children’s ASD symptoms or the amount of (in)formal support that parents received during the COVID-19 lockdown may have influenced levels of parental well-being as well as children’s EB-problems.

Given that, in particular, children with ASD showed great interindividual variability in their responses to the COVID-19 lockdown, it is important that clinicians provide adjusted and personalized support (Spain et al., 2021). For instance, children who deteriorated in functioning may benefit from continuity of daily routines (e.g., going to school) and of access to adequate mental health services (e.g., virtual therapy, parental support) during lockdown periods and other life-changing circumstances (Cost et al., 2022; Kalvin et al., 2021; Spain et al., 2021). Likewise, social skills interventions can support children with ASD, including children who showed improved functioning during lockdown, during the return to school and in habituating to social environments post pandemic (Spain et al., 2021). Moreover, our finding that children with ASD had elevated levels of EB-problems highlights the need for effective intervention strategies such as cognitive behavioral therapy (Kreslins et al., 2015) or parent training (Deb et al., 2020) to reduce problem behavior and optimize the well-being of children with ASD and their parents.

Although this study employed a rigorous methodology, including a pre-registered study plan, a baseline assessment before the COVID-19 pandemic, and a matched sample to reduce bias due to confounding variables, some limitations should also be noted. First, due to a low response rate of children with ASD during COVID-19, child EB-problems were based on parent report in the ASD sample, whereas child report was used in the control sample, which might have affected the comparability of the samples. Self-report instruments can provide valuable information about children’s feelings and behavior, however, this can be challenging in children with ASD, especially when lower IQ levels prevent the valid use of self-report measures (Bakhtiari et al., 2021). Although ASEBA questionnaires (i.e., CBCL, YSR, BPM) have shown moderate to good cross-informant agreement in both general and ASD populations (e.g., Achenbach et al., 2011; Hurtig et al., 2009), it cannot be ruled out that parents and children have different views on emotional and behavioral functioning. The chance that the current results are affected by informant discrepancies should be acknowledged. In future studies, it would be valuable to compare the impact of stressful circumstances on EB-problems between children with and without ASD by using both respondents jointly. Second, indicators of parental mental health and social connectedness were assessed during the COVID-19 pandemic. Parents of children with ASD experienced increased stress and anxiety during the pandemic (Yilmaz et al., 2021), and emerging evidence indicates that this distress can *spill over* to their children, leading to increased EB- problems (Eshraghi et al., 2022; Russell et al., 2020). This suggests that the well-being of parents during the lockdown may be particularly important for their children’s functioning. Nevertheless, the possibility that these self-reports of the parental characteristics had already been affected by the lockdown (Polónyiová et al., 2022) or by the behavioral difficulties of their children (Falk et al., 2014), can be considered a limitation of this study. Clinical data from prior to the pandemic was available on child functioning, allowing for a direct comparison of behavior before and during the pandemic. However, pre-COVID-19 data did not include parental functioning. Third, we examined both (changes in) emotional and behavioral problems as outcomes during the COVID-19 pandemic, as resilience may depend on the domain of outcome studied (e.g., Infurna & Luthar, 2018). Yet, our study does not yield an all-compassing understanding of children’s resilience in this stressful period, given that we only focused on certain maladaptive outcomes. To get a more comprehensive picture of children’s functioning and resilience in times of a pandemic, we recommend that studies examine adaptive outcomes (e.g., positive affect, self-efficacy) as well.

In conclusion, our findings indicate that the impact of the COVID-19 pandemic in terms of emotional and behavioral changes at group level did not differ significantly between children with and without ASD. In both samples, some children experienced an increase in EB-problems, whereas others remained stable or even seemed to thrive during the lockdown. Especially children with ASD showed large interindividual variability in emotional and behavioral functioning before and during the pandemic. Furthermore, indicators of parental mental health and social connectedness were not associated with resilience of children with ASD in times of COVID-19, at least at group level. These results highlight the need for adjusted and personalized support for children with ASD and their parents during the pandemic and future life-changing circumstances.

## Electronic supplementary material

Below is the link to the electronic supplementary material.

Supplementary Material 1

Supplementary Material 2
