# Supplementary material for: Children with Autism Spectrum Disorder in Times of COVID-19: Examining Emotional and Behavioral Problems, Parental Well-Being, and Resilience
Source: J Autism Dev Disord. 2023 May 22;55(2):752–63. doi: 10.1007/s10803-022-05846-y (PMC10201036; doi:10.1007/s10803-022-05846-y)
Supplement: Supplementary file 1 — Supplementary Material 1 [file 10803_2022_5846_MOESM1_ESM.pdf]

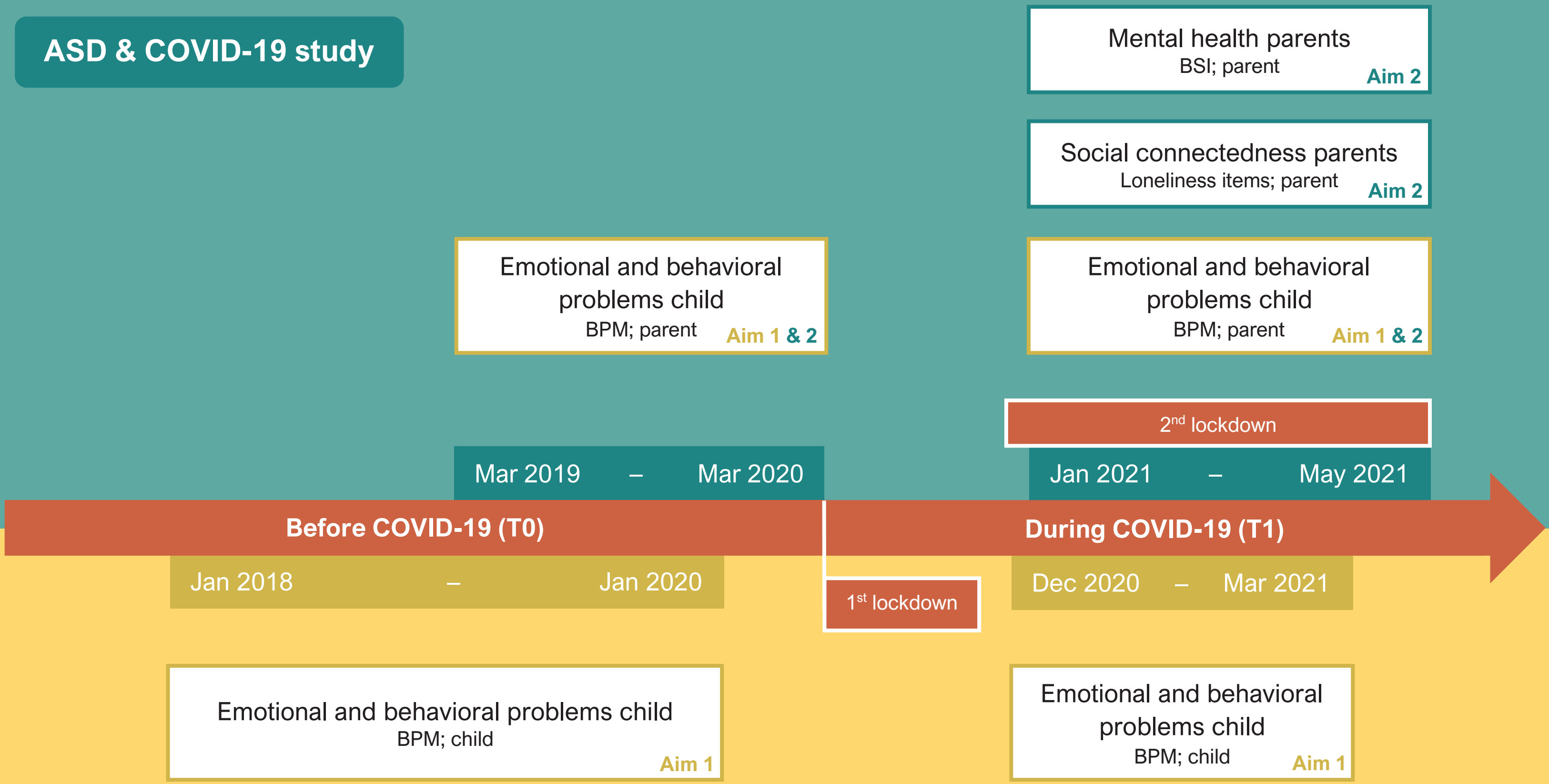

Supplementary material B

Figure S1. Study variables, measures, and timeline of the two study samples
