# Supplementary material for: Children with Autism Spectrum Disorder in Times of COVID-19: Examining Emotional and Behavioral Problems, Parental Well-Being, and Resilience
Source: J Autism Dev Disord. 2023 May 22;55(2):752–63. doi: 10.1007/s10803-022-05846-y (PMC10201036; doi:10.1007/s10803-022-05846-y)
Supplement: Supplementary file 2 — Supplementary Material 2 [file 10803_2022_5846_MOESM2_ESM.docx]

**Supplementary Material**

**Second COVID-19 Lockdown in the Netherlands**

During the second lockdown in the Netherlands, primary schools and day care centers were closed (14 December 2020 – 8 February 2021), as well as secondary schools (14 December 2020 –1 March 2021). Additional governmental measures included: remote working, social distancing, and a curfew. For more details on the timeline, see <https://www.rivm.nl/gedragsonderzoek/tijdlijn-maatregelen-covid>.

**Missing Data**

**ASD Sample:** Little's MCAR test revealed that missing values on study and background variables (23.6%) were missing completely at random (χ2 (170) = 194.04, *p* = .100). Missing data on emotional and behavioral problems (EB-problems) at T0 and T1, Δ EB-problems, parental mental health at T1, social connectedness at T1, and child IQ were handled by performing multiple imputation using 40 imputed datasets and 60 iterations in SPSS version 26.0 (IBM Corp., 2019). We used information on EB-problems at T0 and T1, Δ EB-problems, parental mental health at T1, social connectedness at T1, and auxiliary variables (i.e., children’s age, sex, and IQ, number of siblings, parental age, marital status) as predictors in the imputation model (Wulff & Jeppesen, 2017).

Results from attrition analyses showed that families included (*n* = 62) did not differ from families not included (*n* = 7) in the analyses regarding background characteristics including child age (*t* (67) = 1.65, *p* = .104), child IQ (*t* (54) = 0.36, *p* = .720), the distribution of child sex (χ2 (1) = 2.35, *p* = .125), marital status (χ2 (1) = 0.38, *p* = .537), or parental education level (χ2 (2) = 0.63, *p* = .729).

**Generation R Sample:** The Generation R sample had complete data on study variables. Missing data on background variables was low (2.8%) and were not missing completely at random (χ2 (132) = 223.50, *p* < .001). Missing values on child IQ were imputed, as child IQ was considered for inclusion as a covariate in the analyses. We used information on EB-problems at T0 and T1, Δ problems, and auxiliary variables (i.e., children’s age and sex, number of siblings, parental age, marital status) as predictors in the imputation model (Wulff & Jeppesen, 2017).

Results from attrition analyses showed that adolescents in the matched, included sample (*n* = 213) did not differ from adolescents that were not included (*n* = 4,294) in the analyses regarding parental age (*t* (223) = 1.85, *p* = .066), parental education level (χ2 (2) = 0.02, *p* = .991), or Δ problems (*t* (492) = 1.96, *p* = .051). Yet, adolescents in the matched, included sample were slightly younger (*t* (4505) = 2.08, *p* = .037), had higher IQ levels (*t (*4012) = -2.04, *p* = .041), and lower levels of EB-problems at T0 (*t* (239) = 3.90, *p* < .001) and T1 (*t* (492) = 3.57, *p* < .001), compared to those in the excluded sample. Furthermore, the matched sample contained relatively more boys (χ2 (1) = 55.85, *p* <.001) and more parents that were single/not living together (χ2 (1) = 11.63, *p* < .001).

**Matching Procedure**

To compare the ASD and Generation R samples, we created a matched dataset using propensity score matching in R (R Core Team, 2017). This technique matches the two samples based on measured covariates to balance the distribution of covariates across samples. The package *MatchThem* (Pishgar et al., 2020) was used to match the multiply imputed datasets. The matching procedure specifications followed the within datasets approach (using Rubin’s rules) and optimal full matching, which matches every unit from the ASD sample to at least one control and every control to at least one unit from the ASD sample (Hansen 2004; Stuart & Green 2008). Eligible for inclusion were 275 children (62 from ASD sample, and 213 from Generation R). We matched the samples on child sex and parental age. Matching on additional covariates (EB-problems at T0 and parental education level) did not result in acceptable balance. Balance on covariates was evaluated using the standardized mean difference (SMD; < 0.25) and variance ratios (< 2.00; Rubin, 2001; Stuart et al., 2013). SMD values (0.04­­­–0.11) and variance ratios (1.18–1.32) indicated adequate balance and fruitful matching on child sex and parental age.

Supplementary Material of: *“Children With Autism Spectrum Disorder in Times of COVID-19: Examining Emotional and Behavioral Problems, Parental Well-Being, and Resilience”;* Journal of Autism and Developmental Disorders
D. A. de Maat^1^, R. Van der Hallen, P. F. A. de Nijs, K. Visser, D. Bastiaansen, F. L. Truijens, E. H. M. van Rijen, W. Ester, P. Prinzie, P. W. Jansen, & L. P. Dekker

^1^ Department of Psychology, Education, and Child Studies, Erasmus University Rotterdam, Rotterdam, The Netherlands; [demaat@essb.eur.nl](mailto:demaat@essb.eur.nl)

**References**

Hansen, B. B. (2004). Full matching in an observational study of coaching for the SAT.

*Journal of the American Statistical Association, 99* (467), 609–618. <https://doi.org/10.1198/016214504000000647>

IBM Corp. (2019). *IBM SPSS Statistics for Windows, Version 26.0.* IBM Corp.

<https://www.ibm.com/analytics/spss-statistics-software>

Pishgar, F., Greifer, N., Leyrat, C., & Stuart, E. (2020). *MatchThem:: Matching and*

*Weighting after Multiple Imputation.* arXiv preprint arXiv:2009.11772.

R Core Team (2017). *R: A language and environment for statistical computing.* R Foundation

for Statistical Computing, Vienna, Austria. <https://www.R-project.org/>

Rubin, D. B. (2001) Using propensity scores to help design observational studies: application

to the tobacco litigation. *Health Services & Outcomes Research Methodology, 2,* 169–188. <https://doi.org/10.1023/A:1020363010465>

Stuart, E. A., & Green, K. M. (2008). Using full matching to estimate causal effects in

nonexperimental studies: Examining the relationship between adolescent marijuana use and adult outcomes. *Developmental Psychology, 44*(2), 395–406. <https://doi.org/10.1037/0012-1649.44.2.395>

Stuart, E. A., Lee, B. K., & Leacy, F. P. (2013). Prognostic score–based balance measures

can be a useful diagnostic for propensity score methods in comparative effectiveness research. *Journal of Clinical Epidemiology, 66*(8), S84–S90. <https://doi.org/10.1016/j.jclinepi.2013.01.013>

Wulff, J. N., & Jeppesen, L. E. (2017). Multiple imputation by chained equations in praxis:

Guidelines and review. *Electronic Journal of Business Research Methods, 15*(1), 41–56. <http://www.ejbrm.com/volume15/issue1>
